# Chimney stove intervention – ready for scale up? CON

**DOI:** 10.1136/thoraxjnl-2016-208318

**Published:** 2016-03-10

**Authors:** Kevin Mortimer

**Keywords:** COPD Pathology, Paediatric Lung Disaese, Pneumonia, Tobacco and the lung

The Global Burden of Disease Study estimated that in 2010 there were 6.3, 3.5 and 3.2 million deaths attributable to tobacco smoking, household air pollution from solid fuels and ambient particulate matter pollution, respectively.^1^ The inhalation of polluted air is the leading risk factor for death and disability globally.

The adverse effects of these three categories of air pollution impact most severely on the vulnerable and poor. This is especially true for household air pollution from solid fuels, which almost exclusively affects the poor in the world's poorest countries.^2^

Around the world approximately three billion people use dirty burning solid fuels like animal dung, crop residues and wood for their day-to-day household energy needs. These fuels are typically burned in open fires for cooking, heating and lighting in or near the home environment. When burned in this way they emit substantial quantities of partial products of combustion like black carbon and carbon monoxide which are harmful to health. The 3.5 million deaths attributable to household air pollution each year are mainly from cardiovascular disease and COPD in adults and pneumonia in young children.^3^ ^4^

In *Thorax*, Heinzerling *et al*^5^ describe the CRECER study, a prospective cohort study of the effects of woodsmoke exposure on children in Guatemala in relation to the introduction of a chimney stove intervention. CRECER builds on the Randomized Exposure Study of Pollution Indoors and Respiratory Effects (RESPIRE) trial that evaluated the effect of a chimney stove intervention on the incidence of pneumonia in young children.^6^ RESPIRE in turn built on decades of painstaking work by the senior authors of the CRECER paper and their collaborators. This is noteworthy from the perspective of appreciating the value of the CRECER paper.

The key finding of CRECER is that delayed installation of a chimney stove intervention is associated with poorer lung growth (assessed using six monthly PEF and FEV1 measurements over an average of 1.3 years) than immediate stove installation. The authors recommend additional studies that include longer follow-up and cleaner stoves or fuels. An alternative view is that it is time to scale up and widely implement chimney stoves on health grounds, which I will argue against. The solution lies instead with the availability of clean air for all to breathe.

Why not chimney stoves? The main reason is that there is insufficient evidence that they are effective and cost-effective as health interventions. Although RESPIRE was a giant leap forward in many respects, the incidence of pneumonia in children (primary trial outcome) was not statistically significantly reduced in the chimney stove group.^6^ CRECER certainly adds evidence that chimney stoves may provide health benefits but it does not provide definitive data (note the non-significant finding in relation to FEV1) and it does not provide cost-effectiveness data that would allow policy and decision makers to weigh this intervention against other key health priorities in low-middle income countries. Furthermore, chimney stoves are by and large no more efficient than open fires; they simply vent emissions to the outdoor environment. It is notable that approximately 500 000 of the deaths in the Global Burden of Disease Study attributable to ambient particulate matter pollution are actually attributable to household air pollution-vented outdoors.^1^ Chimney stoves consume more or less the same amount of fuel as open fires and so contribute in the same way to local environmental destruction and adverse health effects associated with this. By emitting the same burden of climate changing black carbon, carbon dioxide and other partial products of combustion they contribute in the same way to global climate change and the adverse health effects associated with this.

Cleaner biomass-burning cookstoves have been developed that emit considerably less carbon monoxide and particulate matter per unit of cooking energy delivered compared with open fires and as such offer promise as health interventions.^7^ The United Nations Foundation Global Alliance for Clean Cookstoves (http://cleancookstoves.org) is a public–private partnership to “save lives improve livelihoods, empower women, and protect the environment by creating a thriving global market for clean and efficient household cooking solutions”. The Alliance has a target of facilitating the adoption of 100 million clean cookstoves to households by 2020. Major global efforts are now underway to scale up the availability of cleaner cookstoves although definitive data about how clean such stoves need to be to deliver health benefits and the cost-effectiveness of such approaches are lacking.

A small number of clinical trials of cookstove interventions are underway that will provide some evidence here. The Cooking And Pneumonia Study is a village level cluster randomised controlled trial of a fan-assisted biomass burning cookstove compared with continuation of traditional cooking methods on pneumonia in children under the age of 5 in rural Malawi (http://www.capstudy.org). The Ghana Randomised Air Pollution and Health Study is evaluating a biomass cookstove and liquefied petroleum gas against traditional cooking methods in 1225 maternal infant pairs on birth weight (http://www.kintampo-hrc.org/projects/graphs.asp).

The bigger picture message I would take from CRECER in the context of the burden of disease caused by the inhalation of polluted air is that lungs everywhere need clean air to breathe. Although we do not yet have definitive evidence on which to base recommendations about specific cookstove interventions on health grounds we do have guidance about how clean air should be including the recently published WHO Indoor Air Quality Guidelines about household fuel combustion.^2^ Chimney stoves, cleaner burning biomass stoves, other types of stoves and cleaner fuels may all be part of the solution but in isolation may not lead to sufficiently clean air to deliver health benefits.

The Sustainable Energy for All (http://www.se4all.org) partnership was launched in 2011 by UN Secretary General Ban Ki-moon to ensure universal access to modern energy services by 2030. This partnership will help drive global efforts to deliver on goal 7 of the post 2015 sustainable development agenda to ensure access to affordable, reliable, sustainable and modern energy for all. To quote Ban Ki-moon “saving our planet, lifting people out of poverty, advancing economic growth—these are one and the same fight.” Research on how to lift people out of poverty of access to clean energy while providing clean air for all to breathe is also part of this same fight.
